# A Waking Review: Old and Novel Insights into the Spore Germination in *Streptomyces*

**DOI:** 10.3389/fmicb.2017.02205

**Published:** 2017-11-13

**Authors:** Jan Bobek, Klára Šmídová, Matouš Čihák

**Affiliations:** ^1^Institute of Immunology and Microbiology, First Faculty of Medicine, Charles University, Prague, Czechia; ^2^Chemistry Department, Faculty of Science, Jan Evangelista Purkyně University in Ústí nad Labem, Ústí nad Labem, Czechia; ^3^Institute of Microbiology of the Czech Academy of Sciences, Prague, Czechia

**Keywords:** dormancy, germination, *Streptomyces*, spore, cell wall, gene expression, metabolism, signaling

## Abstract

The complex development undergone by *Streptomyces* encompasses transitions from vegetative mycelial forms to reproductive aerial hyphae that differentiate into chains of single-celled spores. Whereas their mycelial life – connected with spore formation and antibiotic production – is deeply investigated, spore germination as the counterpoint in their life cycle has received much less attention. Still, germination represents a system of transformation from metabolic zero point to a new living lap. There are several aspects of germination that may attract our attention: (1) Dormant spores are strikingly well-prepared for the future metabolic restart; they possess stable transcriptome, hydrolytic enzymes, chaperones, and other required macromolecules stabilized in a trehalose milieu; (2) Germination itself is a specific sequence of events leading to a complete morphological remodeling that include spore swelling, cell wall reconstruction, and eventually germ tube emergences; (3) Still not fully unveiled are the strategies that enable the process, including a single cell’s signal transduction and gene expression control, as well as intercellular communication and the probability of germination across the whole population. This review summarizes our current knowledge about the germination process in *Streptomyces*, while focusing on the aforementioned points.

## Introduction

Soil microorganisms are exposed to periodic nutrient exhaustions and various abiotic and biotic stresses that inhibit growth. An important survival strategy for many bacteria and fungi in the face of such physiological stresses is the cells’ transition into a dormant state. In the dormant state, cells arrest their growth, discontinue replication and transform themselves into metabolically inactive (or with limited activity), widely resistant forms. Several bacterial clades living in soil, such as *Actinomyces, Streptomyces*, and *Micromonospora*, differentiate into dormant fungi-like uninucleoid spores (arthrospores or exospores). Arthrospores significantly differ from the endospores of *Bacilli* and *Clostridia* in morphology and function. The endospores exhibit striking resistance to a wide range of environmental stresses, such as heat, desiccation, and ultraviolet radiation (Setlow, 2007; Galperin et al., 2012). Their abilities to survive harsh conditions result from multi-layered surface structures and extremely low water content (Setlow et al., 2006; Henriques and Moran, 2007). The well-studied endospores of *Bacillus subtilis* contain high levels of dipicolinic acid in chelation with divalent cations (Ca^2+^) and extremely stable small spore proteins (Setlow et al., 2006). All these features contribute to the overall robust endospore resistance. The streptomycete arthrospores, on the other hand, are more reminiscent of the spores of eukaryotic fungi, possibly due to their convergent evolution in the soil environment. *Penicillium* species, for example, produce conidiophores (analogous to streptomycete aerial hyphae, see below) that bear individually constricted conidiospores (Foster et al., 1945). Both, streptomycetes and molds produce large numbers of small hydrophobic spores with similar properties; both contain, for example, a relatively thick coat (Briza et al., 1990; Pammer et al., 1992; Neiman, 2005), protective small molecules including sugars (such as trehalose, see below), and heat shock proteins (Wyatt et al., 2013). Spores of these organisms not only guard genetic information during unfavorable conditions, but are also adapted to wind dispersal and may remain airborne for long periods.

Their complex life cycle and the described similarities with eukaryotic fungi make streptomycetes unique organisms within the bacterial kingdom. However, comprehensive information about the diverse features of spore germination in this antibiotic-producing clade is still missing. This review attempts to fill this gap.

The process of germination, when either considering the germination rate of a single spore or the probability of germination within a spore population, varies between different *Streptomyces*. Some species (mainly *S. viridochromogenes* and *S. granaticolor*) exhibit fast and robust germination with nearly all spores germinating. That is also why this organism was subjected to many initial germination experiments (Hirsch and Ensign, 1976a,b; Mikulik et al., 1977; Bobek et al., 2004; Xu and Vetsigian, 2017). In contrast, other species (e.g., *S. coelicolor* and *S. venezuelae*) germinate more slowly with a fraction of spores that does not germinate at all. These exhibit more complex germination behavior and possess a fraction of germlings that stop growing soon after germination, probably due to produced inhibitory compounds, such as germicidins and hypnosins, that affect their development (Petersen et al., 1993; Aoki et al., 2007, 2011; Ma et al., 2017; Xu and Vetsigian, 2017). Over time, *S. coelicolor* became a widely used model streptomycetes whose genome was the first to be resolved and best annotated within the genus (Bentley et al., 2002). Therefore, the later genome-wide expression analyses of germinating spores have also been conducted on this stain (Strakova et al., 2013a,b, 2014; Bobek et al., 2014). Hence, most of the findings mentioned here come from the experimental results obtained with *S. coelicolor*.

## The *Streptomyces* Cell Cycle and Sporogenesis

Our knowledge of the complex life of *Streptomyces* with a certain emphasis on the process of sporulation and spore maturation (together here called as sporogenesis) offers many clues for understanding the readiness of spores for successful germination. The life cycle of *Streptomyces*, similarly as in the other arthrospore-forming bacteria, encompasses a development of a network of branched hyphae that grow into the substrate thus creating a vegetative mycelium. The subsequent development of aerial hyphae and spores is considered to be the cell’s response to nutrient depletion. The sporulation-specific regulon is activated after repression of sporulation-specific genes by the BldD-c-di-GMP complex is released (Tschowri et al., 2014; Bush et al., 2015). A part of the vegetative mycelium lyses to be used as a surrogate of nutrients. During this developmental phase the activity of secondary metabolism reaches its maximum to synthesize various bioactive compounds, including antibiotics, in its effort to avoid competitive organisms.

The aerial mycelium of *Streptomyces* is covered with a rodlet layer, a network of pairwise amyloid fibrils (Wildermuth et al., 1971) that consist of chaplin and rodlin proteins (Claessen et al., 2004). This hydrophobic structure lowers the water surface tension thereby enabling the hyphae to grow apically into the air (Elliot and Talbot, 2004; Claessen et al., 2006). The erected sporogenic hyphae are dissected by sporulation septa. The sporulation septation is synchronized with a segregation of chromosomes thus forming unigenomic pre-spore compartments (Ausmees et al., 2007; Wang et al., 2007; Jakimowicz and van Wezel, 2012; Ditkowski et al., 2013). This process is controlled by a family of SsgA-like proteins (SALP) found exclusively in differentiating actinomycetes (Sigle et al., 2015). Their member SsgA is a protein essential for sporulation in *S. coelicolor*; in concert with SsgB it dynamically controls the assembly of FtsZ rings at septation sites (van Wezel et al., 2000; Keijser et al., 2003; Sevcikova and Kormanec, 2003; Willemse et al., 2011). The role of SsgA is related to the *de novo* peptidoglycan synthesis; SsgA has been suggested to mark future germination sites and when the protein is overproduced, individual spores generate many germ tubes. SsgC, whose deletion leads to hypersporulation, has been suggested to function as an antagonist of SsgA. The SALP proteins also include SsgD that ensures the development of the thick spore wall and SsgG that controls regular localization of division sites (Noens et al., 2005, 2007; Traag and van Wezel, 2008).

The following process of spore maturation is the transition of the dissected aerial hyphae into the mature ovoid spores. Not much is known about this phase. When spores are only partially mature (harvested at an earlier stage of sporulation), they germinate remarkably faster and more synchronously. On the other hand, the fully maturated spores exhibit less viability and longer lag-phase in conditions suitable for germination (Hirsch and Ensign, 1976a). These differences hint at further but not yet fully understood processes associated with spore maturation, presumably involving the stabilization of nucleic acids and proteins, deactivation of the metabolic apparatus, desiccation, and changes in the cell wall structure. The spore’s wall thickening is dependent on the so called *Streptomyces*-spore-wall-synthesizing complex (SSSC), a system somewhat similar to the elongasome of rod-shaped bacteria (Kleinschnitz et al., 2011). Members involved in this complex are the cytoskeletal actin-like proteins, MreB and Mbl that cooperate in spore wall synthesis (Mazza et al., 2006; Heichlinger et al., 2011). Maturation is further accompanied by DNA condensation and spore pigmentation, such as the production of gray spore pigment in *S. coelicolor* (Kelemen et al., 1998). After maturation, the rodlet layer forms a thin, basket-like fibrous sheath around the thickened spore wall (Claessen et al., 2002, 2003; Elliot et al., 2003) and cell wall hydrolases under control of other SALP members, SsgE and SsgF, separate individual spores allowing for their dispersal into the environment (Traag and van Wezel, 2008; Flardh and Buttner, 2009; Haiser et al., 2009).

## Dormant Spore Equipment

Dormant spores of streptomycetes are the only haploid state in the development of this multicellular mycelial bacterium. Their main function is to safeguard genetic information throughout unfavorable conditions and consequently spread it into new niches. All the macromolecules that are needed for the future launch of germination have to be pre-synthesized before dormancy. Yet, it remains unclear whether dormant spores possess none or limited metabolism. Despite the commonly held belief that dormant spores are metabolically inactive, Liot and Constant suggested some metabolic activity of dormant spores as their experiments showed that mature spores of *S. avermitilis* (suspended in Tris-HCl) are able to oxidize atmospheric H_2_ to supply maintenance energy (Constant et al., 2008; Liot and Constant, 2016). A fascinating example of metabolically active spores comes from another soil-inhabiting actinomycete genus, *Actinoplanes*, which produces flagellated spores (zoospores) that guided by chemotaxis (sugars, amino acids, aromatic compounds, and mineral ions) are temporarily able to rapidly swim (Palleroni, 1976; Jang et al., 2016).

Generally, other than the thick cell wall, the lack of water in spores ensures their resistance to thermal extremities and to other physical and chemical effects (Kalakoutskii and Agre, 1976). On the other hand, the dehydrated state results in immobility and changed conformation of macromolecules; the condensation of nucleic acids and an inactive form of proteins. The endospores of *Bacilli* contain a significant amount (20%) of dipicolinic acid (Setlow et al., 2006) that stabilizes their macromolecule structures under dehydrated conditions. In the spores of streptomycetes that lack the Ca-dipicolinate, the stabilization and protection of macromolecules is provided by trehalose, instead. During spore resuscitation, the protective role is transferred to present protein chaperones that help other proteins reobtain their functional conformation (Bobek et al., 2004).

Spores subjected to germination in the presence of the antibiotic rifampicin that inhibits the initiation of transcription were still able to synthesize proteins (termed early proteins) over the experimental period. This result suggested that there is also a pool of stable mRNAs, functional ribosomes, and translational apparatus that have been preserved during spore maturation (Mikulik et al., 2002).

In addition, preserved hydrolytic enzymes are accessible for the initial reconstruction of the cell wall (Haiser et al., 2009). In *S. coelicolor*, several hydrolases that might take their part in the cell envelope reconstruction (SCO1061, SCO1725, SCO3487, and SCO5466) were shown to be stored throughout spore dormancy and not synthesized *de novo* during germination (Strakova et al., 2013a). This suggests that cells possess the required enzymatic equipment from the sporulation and/or spore maturation period.

While common opinion has, until recent times, stipulated that spores require external nutrients to launch germination (Flardh and Buttner, 2009), *B. subtilis* spores were recently shown to germinate even in an environment lacking all germination factors (Sturm and Dworkin, 2015; van Vliet, 2015). The launch of germination was stochastic (discussed below) and depended on the level of a transcription factor involved in spore assembly. In the case of *Streptomyces*, the probability of germination was suggested to be dependent on an insoluble protein NepA (de Jong et al., 2009). NepA is a structural part of the streptomycete spore wall which was shown to maintain the spore’s dormancy. In the absence of this protein spores germinate faster and more synchronously. Even spores lacking NepA protein were able to produce hyphae (after 3 days) and form mycelial clumps (after 7 days of cultivation) when cultivated in water (de Jong et al., 2009). This would not be possible if the spores did not possess intracellular nutrient sources, such as trehalose (Ranade and Vining, 1993) and polyphosphates (volutin) (Ghorbel et al., 2006; Strakova et al., 2013b) that promote the metabolic restart before the external sugar can be sensed and assimilated. Various strains of *Streptomyces* have been reported to contain high trehalose levels in their spores. For example in the glucose excess, spores of *S. griseus* accumulate trehalose that comprises up to 25% of their dry weight (McBride and Ensign, 1987a). Trehalose is also abundant in *S. venezuelae* spores, whereas glycogen and polyhydroxybutyrate are absent, which stands in contrast to vegetative hyphae (Ranade and Vining, 1993). It has been shown that sporogenic hyphae convert present glycogen to trehalose during the final period of spore maturation in *S. brasiliensis* (Rueda et al., 2001).

Trehalose is a disaccharide consisting of two glucose molecules that are bound together in α, α-1,1-glycosidic linkage. Trehalose associates in clusters resulting in a large, continuous aggregates formation (Sapir and Harries, 2011). According to the water displacement theory, trehalose present in a system replaces water under desiccation conditions (Sola-Penna and Meyer-Fernandes, 1998). Trehalose is present in various organisms; from Archaea to animals where it may serve as a source of carbon and energy or act as a signaling molecule (Elbein et al., 2003). The unique α, α-1,1-glycosidic linkage makes trehalose non-reducing and therefore unreactive, making it the best intracellular macromolecules stabilizer. Thanks to its ability to make hydrogen bonds with membranes and nucleic acids, as well as its ability to modify the solvation layer of proteins (Sola-Penna and Meyer-Fernandes, 1998), trehalose is capable of protecting cellular membranes, DNA, enzymes (though in their inactive forms), and even whole microorganisms (Yoshinaga et al., 1997; Kandror et al., 2002; Jain and Roy, 2010). The sugar may thus help the cells cope with heat, freezing, desiccation, radiation, and oxidative stresses (Hottiger et al., 1989; Wiemken, 1990; De Virgilio et al., 1994; Yoshinaga et al., 1997; An et al., 2000; Benaroudj et al., 2001; Fillinger et al., 2001). Its multi-protective role is of special need in dormant stages; the sugar is thought to form a gel phase in anhydrous conditions, which prevents aggregation of macromolecules. Subsequent rehydration allows normal restoration of cell metabolism. Consequently, trehalose, as a protective sugar, is quite abundant in bacterial cells, such as those of *Mycobacterium*, including *M. smegmatis* and *M. tuberculosis* (Elbein and Mitchell, 1973), in spores of *Myxococcus* (McBride and Zusman, 1989) and *Streptomyces* (Martin et al., 1986), or in spores of eukaryotic yeast and fungi (Trevelyan and Harrison, 1956; Nwaka and Holzer, 1998). Trehalose is also located inside mycobacterial and corynebacterial cell wall structures (Lederer, 1976).

## The Spores’ Awakening

Minimal or no metabolic activity enables the spores to survive harsh conditions such as high temperatures or the presence of antibiotics and thus stay persevered for years. To awake, spores require at least aqueous conditions (Hirsch and Ensign, 1976b). Germination is faster and more convenient, however, in the presence of required nutrients and other germination stimuli (Paredes-Sabja et al., 2011). Despite this, the scattering remains high in the germination rate within a single population. This being the case, Jyothikumar et al. (2008) showed by means of time-lapse microscopy that germination tubes occurred between 3.75 and 7.5 h in their experimental conditions. The known stimuli that consequently lead to a more synchronous development are heat shock (Hirsch and Ensign, 1976a), mechanical disruption of the spore envelopes (Mikulik et al., 1977; Stastna, 1977; Miguelez et al., 1993), or the presence of peptidoglycan residues (Shah et al., 2008). But how may metabolically inert spores detect optimal environmental conditions? As was shown in the case of the *B. subtilis* spores, germination is launched stochastically, though at much lower frequency in poor environments (Paidhungat and Setlow, 2000; Epstein, 2009; Sturm and Dworkin, 2015). If the environmental conditions remain adverse, for example because of the lack of nutrients, the germinating spore will eventually die (van Vliet, 2015). Thus spores of *Saccharomyces cerevisiae*, an eukaryotic yeast, that launch germination in a poor glucose solution, without any additional nutrients, are not able to further develop bud emergences in these conditions (Chen et al., 2000). In the optimal environment, however, spores may even stimulate each other’s development. In addition to the spore-forming *Streptomyces* (Xu and Vetsigian, 2017) and *Bacilli* (Chen et al., 2006; Sturm and Dworkin, 2015; van Vliet, 2015), the stochastic development from a dormant state was observed in non-sporulating *Escherichia coli* and *M. smegmatis* as well (Balaban et al., 2004; Buerger et al., 2012). Such a strategy where successful pioneers wake other fellows up in a favorable environment is probably advantageous and cost-effective from the perspective of the whole population.

## Germination Stages

**FIGURE 1:**
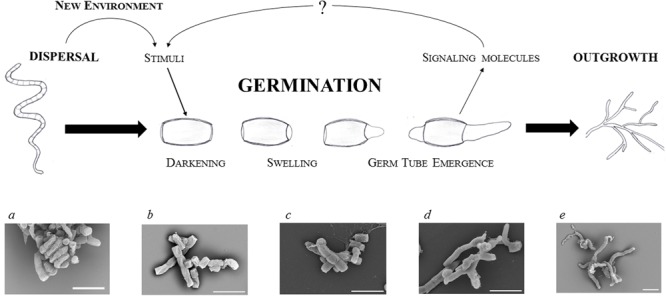
Focus on streptomycete spore germination. Schematic illustration of a germinating spore and electron microscopic images of dormant (a,b) and germinating (c–e) spores of *S. coelicolor*: residue of an aerial hypha with a twisted chain of matured spores (a); dormant spores (b); time-lapse imaging of germinating spores after 2, 4, and 6 h of cultivation (c–e, respectively) in a complex medium (for details see Čihák et al., submitted). The white bars indicate 2.5 μm.

Germination is associated with a complete reconstruction of the cell that involves highly accelerating metabolic activity, morphological changes that start with uncoating, and reconstitution of the cellular content. Spore germination is a sequential process and thus can be divided into three distinctive steps (defined by Hardisson et al., 1978): darkening, swelling, and germ tube emergence (**Figure 1**).

(a) Darkening is associated with cell wall reconstruction
The first step could be described as a transition from pure physical processes, such as osmosis, that progressively activate initial biochemical activities requiring energy sources. During this step spores lose their hydrophobicity resulting in water influx, swelling, and the subsequent loss of heat resistance. These processes change the optical features of spores and allow the cells to re-activate metabolism within the only several minutes after germination initiation. The darkening is caused by the loss of light refraction which requires bivalent cations Ca^2+^, Mg^2+^, Mn^2+^, Zn^2+^, and Fe^2+^ (Hardisson et al., 1978; Eaton and Ensign, 1980; Salas et al., 1983). The binding sites for calcium and magnesium are provided by both, the carboxyl units of peptidoglycan and the polyphosphate groups of teichoic acid in the spore wall (Thomas and Rice, 2014). Relatively early the spores undergo an uncoating, during which calcium accumulated in the spore envelope is gradually released (Eaton and Ensign, 1980; Salas et al., 1983). This process is mediated by a calcium-binding protein CabC whose disruption leads to prematurely germinating spores on the spore chain, while an overexpression of the gene delays their development (Wang et al., 2008). During uncoating, spore lysozyme-like hydrolases are reactivated and facilitate changes in spore morphology and optical features. These enzymes provide lysis and facilitate the reconstruction of cell wall peptidoglycan to allow the entrance of external nutrients. Mutants of two cell wall hydrolases, RpfA and SwlA, exhibited slower germination (Haiser et al., 2009). The hydrolases cleave covalent bonds inside the peptidoglycan layer; an act that must be well-coordinated with the new cell wall synthesis. RpfA belongs to a class of so called resuscitation-promoting factors, Rpf, a subgroup of lysozyme-like transglycosylases, which hydrolyze the beta-(1,4)-glycosidic bond between *N*-acetylglucosamine and *N*-acetylmuramic acid (Mukamolova et al., 1998b, 2002, 2006; Cohen-Gonsaud et al., 2004; Keep et al., 2006; Telkov et al., 2006; Hett et al., 2008; Haiser et al., 2009; Ruggiero et al., 2009). These muralytic enzymes are known to participate in the restoration of active growth from dormancy not only in *Streptomyces* but also in other actinobacteria, like *Mycobacterium* and *Micrococcus* (Mukamolova et al., 1998a, 2006; Keep et al., 2006). *S. coelicolor* possesses five Rpf proteins (RpfA to RfpE) (Haiser et al., 2009) whose individual mutants delay germination to varying extents; with RpfA and RfpE having a stronger effect than RpfC and RpfD. This differs to what can be observed in the wild type (Sexton et al., 2015). Although a multiple *rpf* mutant (with deletion of all five *rpf* genes) revealed a highly impaired germination, the strain was able to germinate within 12 h. This suggests that the Rpf hydrolase family is highly important but not essential for a smooth germination. The Rpfs family could probably be substituted by other cell wall hydrolases. Three *Streptomyces* cell wall lytic enzymes (SwlA to SwlC) are examples of such possible candidates that may be responsible for the proper thickness of the dormant spore wall ensuring its heat resistance (Haiser et al., 2009). In this reference one may find an overview of predicted streptomycete cell wall hydrolase genes as well.
(b) Enzymatic activities are recovered during swelling
Continual influx of water causes spores to swell. A dramatic intracellular decline of the trehalose level has been observed in *S. griseus* during spore swelling (McBride and Ensign, 1987b, 1990). A similar effect was also observed in *M. smegmatis* (Shleeva et al., 2017), *Neurospora* (Sussman and Lingappa, 1959; Sussman, 1961), and *Dictyostelium discoideum* (Roth and Sussman, 1966; Ceccarini, 1967). Consequently, the glucose level increases in swelled spores, suggesting a restored activity of the trehalase enzyme due to an increased concentration of intracellular ATP (McBride and Ensign, 1987b). The hydrolysis of trehalose appears to be an essential step in spore germination (Nwaka and Holzer, 1998). Based on observations of the yeast *S. cerevisiae*, trehalase possesses an inactive form which is activated by phosphorylation in a cAMP-dependent manner (Thevelein, 1984). The restored glucose subsequently provides an internal energy source for the revived enzymes and initial development. Only after the intracellular concentration of trehalose declines do the protected proteins refold into their active forms with the help of their respective chaperones (Singer and Lindquist, 1998).
The hydration of the cytoplasm supports the re-activation of assorted proteins and ribosomes that are preserved from dormancy (Cowan et al., 2003). Bound chaperones GroEL, Trigger factor and DnaK assist in the reactivation of the proteosynthetic apparatus and nascent proteins (Bobek et al., 2004). These chaperones as well as others (GrpE and peptidyl-prolyl *cis–trans* isomerases) are constitutively expressed throughout the germination course suggesting that their presence is constantly necessary (Strakova et al., 2013a). Within several minutes of the germination starting, the ribosomes present are fully functional and new proteins are translated from the stable mRNA stock (Strakova et al., 2013b). The proteosynthesis accelerates between 30 and 60 min (Strakova et al., 2013a). At this stage spores are metabolically active and able to utilize primarily internal energy sources, such as trehalose (see below; Elbein, 1974; Crowe et al., 1984), to obtain carbon, nitrogen and energy (Hey-Ferguson et al., 1973; McBride and Ensign, 1987a). The obtained energy is exploited not only for the intensive proteosynthesis (Mikulik et al., 1984, 2008, 2011; Paleckova et al., 2006) but also for the first DNA replication, which occurs just before or simultaneously with the germ tube emergence within about 60 min of germination in *S. granaticolor* (Mikulik et al., 1977) or 30–60 min later in *S. coelicolor* (Ruban-Osmialowska et al., 2006; Wolanski et al., 2011). Later the spores are able to detect external nutrient sources and as a response they adjust metabolic pathways. They are capable of this due to the activity of present pleiotropic gene expression regulators acting on both transcriptional (BldD, cyclic AMP-receptor protein Crp, and sigma and anti-sigma factors) and translational (ribonuclease RNase III) levels, as was shown in *S. coelicolor* (Strakova et al., 2013a).
(c) The vegetative growth starts with germ tube emergence
The eventual germ tube emergence is a microscopically observable event that provides the basis for the apically growing hyphae. The not yet septated tubes rise from the inner wall of spore (Glauert and Hopwood, 1961) and progress through the outer rodlet layer. An important role is carried out by the chaperonin-like protein SsgA that localizes sites of germ tube emergences. This protein is located at the sites of the germ tip appearance (Noens et al., 2007). In *S. coelicolor* one or two germination tubes appear after 180 min of germination and elongate by apical tip extension. At this developmental point two or three DNA replisomes were demonstrated (Ruban-Osmialowska et al., 2006; Wolanski et al., 2011). The critical determinant of the vegetative growth is the DivIVA protein that localizes at hyphal tips ensuring a new cell wall outgrowth (Flardh, 2003a; Flardh et al., 2012; Hempel et al., 2012; Richards et al., 2012). Interestingly, DivIVA is expressed from early on after spore hydration and its synthesis increases during the whole process of germination (Strakova et al., 2013a). As was also shown, cell division-associated protein FtsZ (SCO2082, Grantcharova et al., 2005), which is the BldD target, and cell growth-associated protein FilP (SCO5396, Flardh, 2003b; Bagchi et al., 2008) are expressed at 300 min of *S. coelicolor’s* germination. FilP protein, which is associated with DivIVA, accumulates in the tips of young hyphae and is also expressed soon after germination starts (Strakova et al., 2013a). FilP resemble eukaryotic intermediate filaments forming a tangled cytoskeletal network that confers to rigidity and elasticity of hyphae (Kelemen, 2017). The onset of these proteins was previously proposed as the point when the germination phase finalizes (Strakova et al., 2013a).

## Proteome Reconstitution and Transcription Control During Germination

Several genome-wide studies on gene expression during the streptomycete spore germination have recently been conducted (Bobek et al., 2004, 2014; Piette et al., 2005; Strakova et al., 2013a,b). Furthermore, a biocomputational modeling (Strakova et al., 2013a,b, 2014) was employed to obtain a global view on protein synthesis and to characterize the regulatory networks. The expression profiles of individual proteins from 13 time points throughout the course of germination have been sorted and divided among differential functional groups. A study analyzing the protein synthesis changes between consecutive time intervals revealed that the transcriptional apparatus is indeed highly active from the first 30 min of germination but surprisingly, most of the mRNAs preserved from dormancy are consequently degraded during the rehydration (Strakova et al., 2013a; Bobek et al., 2014). The degradation affects mRNAs from the majority of functional protein groups, i.e., amino acid, carbohydrate, lipid and energy metabolism, proteasomes, and DNA repair pathways. This does not mean, however, that the activity of these functional groups is disabled. For example, besides those mentioned, the degraded mRNAs also encode proteins involved in translation. This occurs despite the intensive protein synthesis, suggesting that the fully active translational apparatus is available from dormancy and is re-activated very rapidly when the germination starts. Subsequently, but still during the first 30 min of germination, the level of most mRNA groups increases again, but now more likely as a relevant response to the available sources and cell requirements under new conditions.

The activity of the transcriptional apparatus is strictly dependent on the set of sigma factors present in distinctive time points. They direct specific developmental tasks, such as the cell wall reconstitution, protein refolding, or the osmotic stress response, and therefore they must be fully accessible at specific points during germination (Bobek et al., 2014). Thus, SigW sigma factor (SCO0632), whose homolog in *B. subtilis* directs the synthesis of cell wall hydrolases (Helmann, 2006), is already expressed in *S. coelicolor* during the initial rehydration event, suggesting a similar role in the cell wall reconstruction. At 30 min of germination, a set of other sigma factors is activated, including HrdD and the stress responding ECF family sigma factors SigR, SigE, SigD, and SigH. SigR as well as SigH direct cellular responses to osmotic stress (Kormanec et al., 2000; Kelemen et al., 2001; Viollier et al., 2003; Zdanowski et al., 2006; Shu et al., 2009; Kallifidas et al., 2010; Sevcikova et al., 2010). Moreover, the SigR sigma factor, whose regulon comprise DnaK and ClpB chaperones, ensures control over the process of protein re-folding (Kallifidas et al., 2010). SigE possibly takes part in cell envelope reconstruction control, similarly to SigW in the early phase (Paget et al., 1999; Hong et al., 2002). However, the highest increase in its cellular level during germination is exhibited by *S. coelicolor*’s SigQ sigma factor (SCO4908; Bobek et al., 2014), whose regulon is now being studied. Most of the sigma factors mentioned are known to respond to stress conditions. Several members of the stress response apparatus that are involved in the antioxidant defense – superoxide dismutase SodF2 (SCO0999), catalase (SCO0379), and thioredoxins TrxA (SCO3889) and TrxA4 (SCO5419) – were also shown to be expressed soon after spore hydration in *S. coelicolor* (Strakova et al., 2013a). The detoxification system thus reflects the increased metabolic activity. Based on the fact that the stress responding proteins and their regulators (sigma factors) are highly active during germination, it could be proposed that germination itself evokes stress-like conditions, due to the entry of water, oxygen radicals, and salts into the reviving spores.

The ATP level increases approximately 11-fold during germination (Hirsch and Ensign, 1978). Its production is ensured by ATP synthase, whose expression is abolished in the crp^-^ mutant strain (see below). ATP is essential not only as an energy source for the basal metabolism (Eaton and Ensign, 1980) but also for cyclic AMP (cAMP) synthesis (Piette et al., 2005). The cAMP is a signal molecule which accumulates during germination and is bound by the cAMP-receptor protein Crp, an important transcription regulator (Susstrunk et al., 1998; Derouaux et al., 2004). Both *Streptomyces* mutant strains in which either *cya* (a gene encoding adenylate cyclase that catalyzes conversion of ATP to cAMP) or the *crp* gene were deleted exhibited a similar, dramatic defect in germination. The germination defect caused by the missing or inactive Crp system can be explained by the subsequent absence of several members of the affected Crp regulon that play a crucial role in germination. Other than ATP synthase, the Crp regulon encompasses the cell wall hydrolases and chaperones (Piette et al., 2005). Piette et al. (2005) described morphological differences between spores of *S. coelicolor* wild type and its crp^-^ mutant strains. The spore wall of the crp^-^ strain was twice as thick as that of the wild type strain. This can be related to the suppressed expression of another cell wall hydrolase, SCO5466, in the crp^-^ strain during germination. Besides SCO5466, the expression of the above mentioned RpfA hydrolase is also under regulation of Crp (St-Onge et al., 2015). One may assume that the Cya-cAMP-Crp system might control the muralytic activity of hydrated spores, among other processes, allowing for cell wall reconstruction; an indispensable procedure for successful germination. A differential protein expression analysis between the wild type and crp^-^ strains, provided by the same authors, revealed decreased expression of protein chaperones GroEL1, GroEL2, DnaK that could have a negative effect on protein folding and thus negatively affect the germination in the crp^-^ strain. The crucial role played by the Cya-cAMP-Crp system in germination may however rely on the aforementioned trehalose hydrolysis control.

## Secondary Metabolism of Germinating Spores

In natural soil environments, germination of spores and seeds from different organisms is affected by a plethora of external factors. These include population density of organisms of the same species, the presence of rival organisms, and respective intra- or inter-species communication. In laboratory conditions, physiological differences, including inhibition of development or degradation of signaling compounds, were observed when *Streptomyces* had been cultivated together with *B. subtilis* or with bacteria containing mycolic acids in their cell walls (Onaka et al., 2011; Traxler et al., 2013).

Germinating spores produce signals that play a role in their own communication framework. The production of an unknown activator that would induce germination under optimal conditions can be expected based on an experiment in which monocultural spores of *S. venezualae* germinated significantly faster in higher spore densities or in a medium supplemented by its own, earlier germlings (Xu and Vetsigian, 2017). On the other hand, a similar experiment provided by the same authors revealed a reduction of the germinating spores at higher spore densities in *S. coelicolor.* The existence of germination inhibitors produced by the germlings was firstly shown in *S. viridochromogenes* (Grund and Ensign, 1985) and later also in *S. coelicolor* (Song et al., 2006). These germination inhibitors include germicidins, gramicidine S, and hypnosin (Ensign, 1978; Grund and Ensign, 1985; Setlow, 2003; Aoki et al., 2007, 2011). At least four germicidin homologs are produced by Gcs, a type III polyketide synthase in *S. coelicolor* (Song et al., 2006). The expression of its gene (SCO7221) highly increases at 30 min of germination, as can be found in the whole genome expression data in Strakova et al. (2013a). The hypnosin, whose genetic substantiality is not clear, is bound to the spore surface and released in wet conditions thus probably preventing premature germination of spore chains on aerial mycelia (Xu and Vetsigian, 2017).

Recent analytical technologies allow more detailed investigations into the production of small molecular compounds. Using high-performance liquid chromatography-mass spectrometry, three different compounds – a sesquiterpenoid antibiotic albaflavenone, the polyketide germicidin A, and the chalcone – were detected to be synthesized upon germination in *S. coelicolor*. The two latter compounds revealed an inhibitory effect on the germination process (Čihák et al., submitted). Germicidin A is known as an autoregulatory germination inhibitor of *Streptomyces* and other bacteria (Petersen et al., 1993). The compound is expected to be a widespread interspecies regulator affecting germination of various sporogenic organisms. Chalcones, as well as other flavonoids and phenolic compounds, are important signaling molecules in plant–microbe symbioses, being essential for the plant survival (Maxwell et al., 1989; Diaz-Tielas et al., 2012). Although *Streptomyces* produce flavonoids [e.g., THN and flaviolin by *S. coelicolor* (Zhao et al., 2005)], the only streptomycete chalcone derivative is known in naringerin biosynthesis, a typical plant secondary metabolite produced by *Streptomyces clavuligerus* (Alvarez-Alvarez et al., 2015). The presence of different germination inhibitors in *S. coelicolor* is in accordance with the fact that the germination rate of this strain is much slower and even decreased with a higher spore densities, as mentioned above.

Several streptomycete antibiotics produced during sporulation, such as neomycin in *S. fradiae*, streptomycin in *S. griseus* (Barabas and Szabo, 1968), or actinorhodin in *S. coelicolor* (Čihák et al., submitted) are known to be bound on the spore envelopes and released into the environment during germination. These antibiotics protect the germinating spores against environmental challenges before the internal biosynthetic pathways are re-activated. Some antibiotics, such as cephamycin C and clavulanic acid both synthesized by *S. clavuligerus* are known to be produced soon after germination (Sanchez and Brana, 1996).

## Perspectives

Spore germination in *Streptomyces* represents an exceptional study model of bacterial cell differentiation that presents a complete transformation of cellular morphology and the restoration of all physiological processes. More detailed knowledge about the structure of dormant spores may provide clues for understanding their readiness to trigger metabolic activity and cell reconstitution.

An interesting area that deserves our attention is the probability of germination and germination control. As discussed in this review, the cell awakening is launched in an aquatic milieu; the probability of awakening differs, possibly depending on the accessibility to nutrients and on other biotic and abiotic factors. Stochastic germination seems to be a widespread strategy of spore-forming organisms that has evolved to ensure the survival of whole populations. From the one-cell perspective, spores represent a safe but non-reproductive state, whereas germination is a risky and irreversible process. From the point of view of the whole population, early germinating pioneers take advantage by occupying vacant niches whereas a hesitant strategy is preferable when conditions become harsh. Therefore, one may assume that the onset of germination is subject to various controlling mechanisms that respond to external signals and activate or suppress the process as is needed. The characterization of these signals and their sensors has not been established to date. The transferred signals are eventually reflected in complete cell reconstruction. This requires the activity of specific genes whose expression is directed by a unique set of transcriptional factors. Characterizing these factors and clarifying their regulons would shed more light on the process by which dormant spores are awakened.

## Author Contributions

JB wrote the whole manuscript, MČ analyzed the knowledge about metabolic activities of germinating spores, KŠ analyzed the knowledge about transcriptional activities of germinating spores.

## Conflict of Interest Statement

The authors declare that the research was conducted in the absence of any commercial or financial relationships that could be construed as a potential conflict of interest.
